# On the Construction, Organization, and General Arrangement of Hospitals for the Insane

**Published:** 1854-11

**Authors:** 


*On the Construction, Organization, and General Arrangement of Hospitals for the Insane.* By Thomas S. Kirkbride, M.D., Physician to the Pennsylvania Hospital for the Insane. Philadelphia: Lindsay & Blakiston, 1854.

The growing interest for the welfare of the Insane manifested by States as well as by individuals, is one of the encouraging signs of the present age. The wants of this unfortunate class of the community are being recognized, while interest and duty alike prompt to measures for their relief. In our own country, institutions are springing up in almost every State, where their physical wants are judiciously cared for, and their mental maladies properly treated. Dr. Kirkbride, in the little work before us, has made some valuable suggestions as to the style of architecture and internal arrangements best suited for the gratification of the taste and the accommodation of the Insane.

We commend the work to the careful perusal of all those interested in the erection or management of hospitals for this class of the community. J.

